# Selective Chemical Vapor Deposition Growth of WS_2_/MoS_2_ Vertical and Lateral Heterostructures on Gold Foils

**DOI:** 10.3390/nano12101696

**Published:** 2022-05-16

**Authors:** Zixuan Wang, Wenshuo Xu, Benxuan Li, Qiaoyan Hao, Di Wu, Dianyu Qi, Haibo Gan, Junpeng Xie, Guo Hong, Wenjing Zhang

**Affiliations:** 1 Institute of Applied Physics and Materials Engineering, University of Macau, Avenida da Universidade, Taipa 999078, Macau; yb87822@umac.mo (Z.W.); jepsenxie@foxmail.com (J.X.); 2 International Collaborative Laboratory of 2D Materials for Optoelectronics Science and Technology of Ministry of Education, Institute of Microscale Optoelectronics, Shenzhen University, Shenzhen 518060, China; bl398@cam.ac.uk (B.L.); hqy@szu.edu.cn (Q.H.); physicswudi@163.com (D.W.); qidianyuqi@sina.com (D.Q.); ganhaibo@szu.edu.cn (H.G.); 3 Department of Physics, National University of Singapore, 2 Science Drive 3, Singapore 117551, Singapore; 4 Electrical Engineering Division, Engineering Department, University of Cambridge, 9 JJ Thomson Avenue, Cambridge CB3 0FA, UK; 5 Department of Physics and Chemistry, Faculty of Science and Technology, University of Macau, Avenida da Universidade, Taipa 999078, Macau

**Keywords:** chemical vapor deposition, 2D materials, heterostructures, selective growth

## Abstract

Vertical and lateral heterostructures consisting of atomically layered two-dimensional (2D) materials exhibit intriguing properties, such as efficient charge/energy transfer, high photoresponsivity, and enhanced photocatalytic activities. However, the controlled fabrication of vertical or lateral heterojunctions on metal substrates remains challenging. Herein, we report a facile and controllable method for selective growth of WS_2_/MoS_2_ vertical or lateral heterojunctions on polycrystalline gold (Au) foil by tuning the gas flow rate of hydrogen (H_2_). We find that lateral growth is favored without H_2_, whereas vertical growth mode can be switched on by introducing 8–10 sccm H_2_. In addition, the areal coverage of the WS_2_/MoS_2_ vertical heterostructures is tunable in the range of 12–25%. Transmission electron microscopy (TEM) and selected area electron diffraction (SAED) results demonstrate the quality and absence of cross-contamination of the as-grown heterostructures. Furthermore, we investigate the effects of the H_2_ flow rate on the morphology of the heterostructures. These pave the way to develop unprecedented 2D heterostructures towards applications in (opto)electronic devices.

## 1. Introduction

Two-dimensional (2D) materials, including graphene, hexagonal boron nitride (h-BN), and transition metal dichalcogenides (TMDs) [1,2,3,4], play a crucial role in advanced devices due to their unique properties [5,6,7,8]. 2D heterostructures consisting of different atomically thin components are formed either through layer-by-layer stacking or in-plane stitching [9,10,11]. The synthetic techniques of transition metal dichalcogenides (TMDs)-based vertical and lateral heterostructures (VHs and LHs) have been studied in recent years, among which chemical vapor deposition (CVD) stands out as a controllable and scalable method. Gong et al. utilized CVD to achieve the selective growth of WS_2_/MoS_2_ VHs and LHs on SiO_2_/Si substrate by varying the temperatures [12]. The mediating role of the growth temperature was further verified by the epitaxial growth of WS_2_/MoS_2_ VHs with reverse stacking sequences on sapphire [13]. In addition to changing the temperature, Zhu et al. controlled the growth of WS_2_/MoS_2_ VHs and LHs by introducing hydroxide molecules after the growth of the bottom layer MoS_2_. This can lead to the formation of −OH groups on the surface of the MoS_2_ at low temperature, thereby hindering the nucleation of the top layer WS_2_ and resulting in WS_2_/MoS_2_ LHs. The decomposition of the −OH at high temperature allows the preferential growth of WS_2_ on top of MoS_2_ and creation of WS_2_/MoS_2_ VHs [14]. The involvement of hydroxides may cause contamination and deteriorate the intrinsic properties of the TMD constituents. Zhang et al. used an additional substrate to confine the growth area of MoS_2_/WS_2_ VHs, and decreased the nucleation density of the top layer WS_2_ on the bottom layer MoS_2_ by introducing H_2_ as an additional carrier gas [15]. Furthermore, Au foils are considered strongly interactive and concurrently compatible with the growth of 2D materials. Both theoretical analysis and experimental observations show that most TMD possess three-fold symmetry and present very similar epitaxial behavior on substrates [13,16,17]. However, the selective fabrication of 2D TMDs-based VHs and LHs on metal substrates has not been investigated, which is important for lowering the contact resistance between the TMD semiconductor channel and the metal electrodes.

In this work, we report the selective growth of WS_2_/MoS_2_ VHs and LHs on polycrystalline Au foil by tuning the gas flow rate of hydrogen. The synthesis approach is facile and the switching of growth mode is achieved via monocontrol. WS_2_/MoS_2_ LHs are preferentially formed without H_2_, whereas H_2_ is necessary for the growth of WS_2_/MoS_2_ VHs Moreover, we study the influence of the H_2_ flow rate on the resultant domain morphology. The effect of substrates is also studied by comparing the growth results on Au, c-sapphire and SiO_2_/Si. Our work will be helpful for the synthesis of unprecedented two-dimensional materials with outstanding properties.

## 2. Materials and Methods

### 2.1. Pre-Treatment of Polycrystalline Au Foils

First, commercially available Au foils (Alfa Aesar, Thermo Fisher Scientific, Ward Hill, MA, USA, 25 μm thickness, 99.985% metal basis, LOT: R23F014) were cut into an appropriate size. Then, small pieces of Au foils were ultrasonically cleaned by an acetone solution and IPA solution for 10 min, respectively. After that, the cleaned Au foils were annealed in the CVD at 1000 °C for 3 h to release the stress and expose the grain boundaries, and Ar with a flow rate of 100 sccm was introduced during the whole annealing process.

### 2.2. CVD Growth Process of LHs and VHs

**Figure 1:**
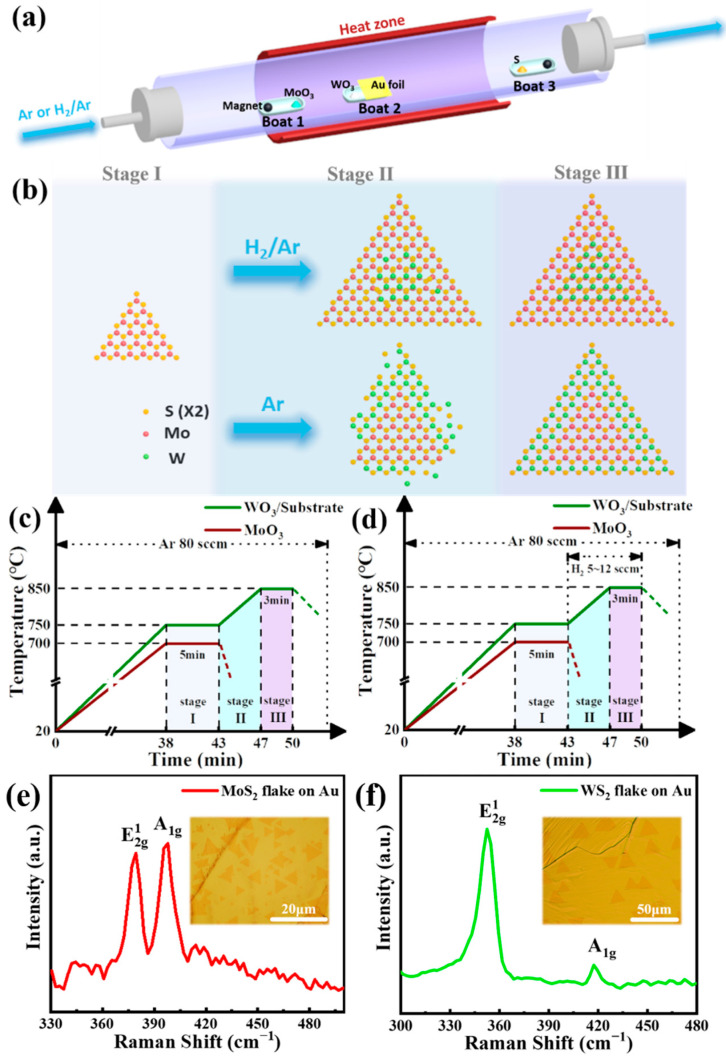
(**a**) Schematics of the CVD setup for the growth of WS_2_/MoS_2_ LHs and VHs; (**b**) schematic illustrations of WS_2_/MoS_2_ LHs and VHs growth process; (**c**,**d**) temperature program of the CVD growth process for WS_2_/MoS_2_ LHs and VHs, respectively; (**e**,**f**) Raman spectra of as-grown MoS_2_ and WS_2_ monolayers, respectively. Insets: Corresponding optical microscopic images of the MoS_2_ and WS_2_ flakes on Au foil.

A chemical vapor deposition device with only one heating zone was shown in Figure 1a. Three boats (named boat 1, boat 2 and boat 3) were placed in quartz tubes from upstream to downstream. Boat 2 with WO_3_ was put in the center of the heating zone, and the Au foil was also placed in boat 2 close to WO_3_ with a distance of about 0.5 cm. Boat 1 with MoO_3_ was put in the left of the heating zone, while boat 3 with S powder was put outside the heating zone in the right. In addition, both boat 1 and boat 3 have one magnet in them to ensure they can be moved during the growth process. The chemical vapor deposition process can be divided into three stages: growth stage Ⅰ was for the first monolayer MoS_2_, and growth stage Ⅲ was for the second monolayer WS_2_ (relatively higher growth temperature), while transition stage Ⅱ was between the two growth stages. Ar was used as the carrier and protective gas with a flow rate of 80 sccm during the whole process.

### 2.3. WS_2_/MoS_2_ LHs Growth on Au Foils

MoO_3_ powder (99.9%, 2 mg) was put into boat 1, WO_3_ powder (99.9%, 3 mg) and Au foil were put into boat 2, while sulfur powder (99.9%, 20 mg) was put into boat 3. In the beginning, WO_3_ was placed at the center of the heating zone. MoO_3_ was placed in the room temperature position in a quartz tube to reduce the vast evaporation, while S was placed downstream at the temperature of about 200–250 °C. Then, the temperature of the heating zone was raised up from room temperature to 750 °C in 38 min and kept at 750 °C for 5 min for the growth of the first monolayer MoS_2_. The Mo source was sent to a 700 °C position in the heating zone (the exact location of 700 °C in the heating zone can be determined by a K type thermocouple thermometer when the heating center reaches 750 °C) as soon as the programmed temperature reached 750 °C, and the distance between Mo source and Au foil was kept at 7.5 cm. Then, boat 1 was pulled out to room temperature to cut off the supply of Mo source after the growth stage Ⅰ. Meanwhile, change the position of boat 3 to a suitable temperature zoom to ensure the S stable supply. Next, the temperature of the heating zone was raised up to 850 °C in 4 min and maintained for 3 min for the growth of WS_2_. Finally, the tube furnace was quickly cooled down to room temperature after the growth stage Ⅲ. The temperature program for WS_2_/MoS_2_ LHs growth is shown in Figure 1c.

### 2.4. WS_2_/MoS_2_ VHs Growth on Au Foils

Similar to the conditions described for WS_2_/MoS_2_ LHs growth above, H_2_ with a flow rate of 5–12 sccm was introduced during the transition stage Ⅱ and growth stage Ⅲ. Keep other experimental parameters and conditions the same as those in WS_2_/MoS_2_ LHs growth. The temperature program for WS_2_/MoS_2_ VHs growth is shown in Figure 1d.

### 2.5. MoS_2_ and WS_2_ Monolayers Growth on SiO_2_/Si and C-Sapphire Substrates

For MoS_2_ monolayers growth, only reserve and extend growth stage Ⅰ to 20 min; for WS_2_ monolayers growth, only reserve and extend growth stage Ⅲ for 20 min. Keep the other parameters and conditions the same as those in the growth case of WS_2_/MoS_2_ LHs.

### 2.6. Transfer Process

After growth, poly methyl methacrylate (PMMA) (10% wt., in anisole) was spin-coated following 2000 rpm for 90 s, and then the sample was baked at 180 °C for 5 min. The PMMA-sample-Au foil was put into the Au etchant solution (I_2_ and KI in a mol ratio of 1:1, dissolved in 50 mL deionized water) at 50 °C for 3 h to remove Au foil. The floating film was transferred into DI water to remove the etchant ions and was finally lifted onto a cleaned SiO_2_/Si substrate. The substrate was then dipped into acetone to remove the PMMA layer.

### 2.7. Characterization

OM images were obtained on an optical microscope (Leica DM2700M RL, Wetzlar, Germany). AFM characterization was performed on a Dimension ICON microscope from Bruker (365 Boston Rd. Billerica MA 01821, USA). Raman spectra, PL spectra, and mappings were collected via Raman spectroscope (Alpha 300, WITec with 532 nm laser, Lise-Meitner-Str. 6 D-89081 Ulm, Germany). The composition and valence of elements were explored by using an XPS (ESCALab250Xi, 81 Wyman Street, Waltham, MA, 02454, USA), and TEM images and element distribution of the heterojunction were analyzed by EDS mapping on a TEM (JEM-3200FS, JEOL, Street No.6, Haidian District, Beijing 100190, China).

## 3. Results and Discussion

WS_2_/MoS_2_ LHs and VHs were fabricated via one-pot CVD. The schematic diagrams of the CVD setup for the growth of the heterostructures the growth process of the VHs are shown in Figure 1a,b, respectively. Distinct from the conventional placements of the precursors, we loaded sulfur powder at the downstream region. This positioning strategy only allows a very small flux of S to get involved in the growth stages by slow diffusion. Therefore, the MoO_3_ is prevented from being exposed to a large amount of S, whereby decreasing the cross-contamination at the resultant heterointerface [18]. The temperature programs for growing the WS_2_/MoS_2_ LHs and VHs are shown in Figure 1c,d, respectively. The growth process can be divided into three stages: Stage Ⅰ represents the growth of the bottom MoS_2_ monolayer, and Stage Ⅲ represents the growth of the top WS_2_ monolayer at a higher temperature, and Stage Ⅱ is a transition stage. Since the boiling point of WO_3_ is higher than that of MoO_3_, the WO_3_ is placed closer to the Au substrate and remains at the same location during the growth. On the contrary, MoO_3_ powder is transited from a room temperature region into the growth area (~700 °C) as soon as the growth program reaches Stage Ⅰ, and returns to the room temperature region to cut off the Mo supply immediately after Stage Ⅰ. More details of the synthesis are described in the Experimental Section. Typical Raman spectra from MoS_2_ and WS_2_ monolayer domains were shown in Figure 1e,f, respectively. The Raman peaks centered at 384.5 and 403.5 cm^−1^ correspond to $E_{2g}^{1}$ and A_1g_ vibrational modes of MoS_2_, while Raman peaks located at 419 cm^−1^ and 355.5 cm^−1^ correspond to $E_{2g}^{1}$ and A_1g_ vibrational modes of WS_2_. $E_{2g}^{1}$ is an in-plane vibration mode, corresponding to the in-plane vibrations of Mo/W and S atoms, while A_1g_ is the out-of-plane vibration mode of the two S atoms along the *z*-axis of the TMD unit cell [5]. Additionally, the frequency differences (Δ) for MoS_2_ and WS_2_ were measured to be 19 and 63.5 cm^−1^, respectively, confirming that the as-grown MoS_2_ and WS_2_ are both monolayer [19]. The insets present the optical microscopic images of the as-grown MoS_2_ and WS_2_ single crystals on Au foils. The lateral sizes of the MoS_2_ and WS_2_ domains are as large as ~10 and ~15 μm, respectively. The growths of the MoS_2_ and WS_2_ domains with similar sizes on SiO_2_/Si and c-sapphire substrates (Figure S1) are much slower (20 min) than those on Au foils (5 min), indicating that Au plays a catalytic role in the synthesis.

Given that the laser exciton would lead to energy transfer from the MX_2_ to the Au, the as-grown WS_2_/MoS_2_ LHs and VHs were transferred onto SiO_2_/Si substrates to further characterize the WS_2_/MoS_2_ heterostructures. The schematic illustration of the transfer process was presented in Figure S2, and the details of the transfer method described in the Materials and Methods section.

**Figure 2:**
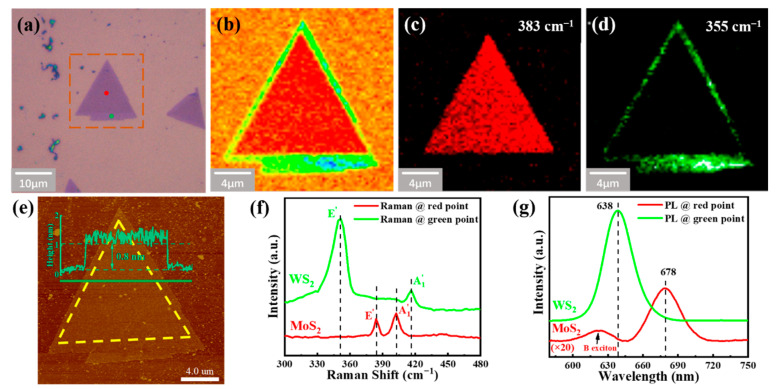
(**a**) Optical microscopic image of an as-grown WS_2_/MoS_2_ LH transferred from Au foil to SiO_2_/Si substrate; (**b**) Raman intensity map; (**c**,**d**) Raman intensity maps at 383 and 355 cm^−1^, respectively. The excitation wavelength is 532 nm; (**e**) AFM image of the WS_2_/MoS_2_ LH acquired from the boxed region in (**a**). Inset: corresponding height profile measured along the green line; (**f**) Raman spectra and (**g**) PL spectra collected at different positions of the WS_2_/MoS_2_ LH shown in (**a**).

Following the growth process in Figure 1f, the WS_2_/MoS_2_ LHs consisting of a MoS_2_ monolayer inside and a WS_2_ monolayer outside were produced. Figure 2a shows the optical image of as-transferred WS_2_/MoS_2_ LH on the SiO_2_/Si substrate. The edge length of the inner MoS_2_ monolayer is about 10 μm. Raman mapping for the area is labeled by a brown dotted square, and the intensity mappings for the Raman characteristic peaks of MoS_2_ (383 cm^−1^) and WS_2_ (355 cm^−1^) are shown in Figure 2b–d, respectively. To measure the thickness of the as-prepared WS_2_/MoS_2_ LH, AFM was carried out. Figure 2e shows the AFM surface morphology and the corresponding height profiles across the whole WS_2_/MoS_2_ LH. The thickness of the sample is about 0.8 nm, thereby proving the monolayer characteristics of the as-synthesized WS_2_/MoS_2_ LH [20]. Additionally, Raman and PL spectroscopy were used to characterize the LH structure. Raman single spectra acquired from the red point shows only the $E_{2g}^{1}$ (at 383 cm^−1^) and A_1g_ (at 402 cm^−1^) peaks of the MoS_2_, and the spectra collected from the green point show only the $E_{2g}^{1}$ (at 355 cm^−1^) and A_1g_ (at 418 cm^−1^) peaks of the WS_2_ (Figure 2f). The difference between the two modes is about 19 cm^−1^ and 63 cm^−1^, respectively, indicating that inside MoS_2_ and outside WS_2_ are both monolayers [12]. On the other hand, the PL spectra collected from the red point show a strong peak and a weak peak at 678 nm and 620 nm, respectively (Figure 2g). The peak at 678 nm is related to the direct transition in the MoS_2_ monolayer [5], proving that the triangular area inside is a MoS_2_ monolayer, and the peak at 620 nm is attributed to the B-exciton because of the energy level splitting [21]. In contrast, the PL spectra collected from the green point show a strong peak only at a wavelength of 638 nm, corresponding to the 1.96 eV direct excitonic transition energy in the monolayer WS_2_ outside. These results confirm the formation of an in-plane WS_2_/MoS_2_ heterostructure, with a triangular monolayer MoS_2_ domain inside and WS_2_ outside.

XPS measurements were also conducted to demonstrate the elemental compositions, and the results are shown in Supporting Information. Figure S3a shows the optical microscopic image of the as-grown WS_2_/MoS_2_ LHs. Figure S3b presents the wide XPS scan collected from the heterojunction area, indicating the existence of Au, W, Mo, and S elements. As shown in Figure S3c–f, respectively, Au 4f (Figure S3c) exhibits two peaks at around 84.2 eV (Au 4f7/2) and 88 eV (Au 4f5/2), which shows the presence of Au (0). The two peaks at around 32.9 and 35.1 eV can be assigned to W 4f7/2 and W 4f5/2, respectively, presenting W (+4) as shown in Figure S3d. There are two major peaks of the Mo element that appeared at around 232.3 (Mo 3d3/2) and 229.5 eV (Mo 3d5/2), respectively, as the presence of Mo (+4) (Figure S3e). Another peak at 226.8 eV corresponds to S 2s (labeled with the blue arrow). Figure S3f shows two peaks at around 163.6 and 162.2 eV, corresponding to S 2p1/2 and S 2p3/2, respectively. All these XPS results are consistent with the values for WS_2_/MoS_2_ heterostructures reported previously [22].

**Figure 3:**
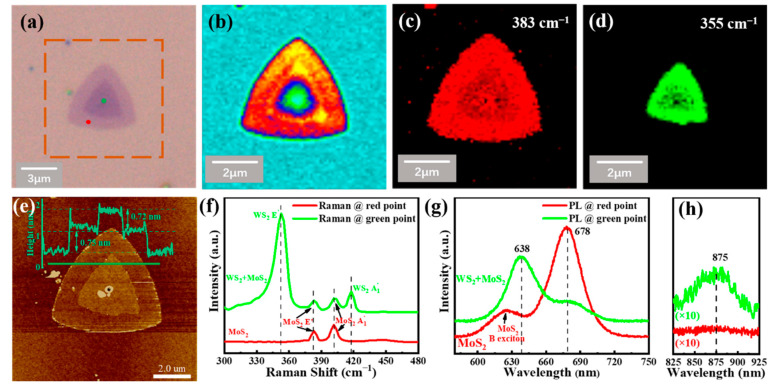
(**a**) Optical microscopic images of the as-grown vertical WS_2_/MoS_2_ heterostructure transfer from Au foil to SiO_2_/Si; (**b**) Raman intensity map; (**c**,**d**) Raman intensity mapping at 383 cm^−1^ (**c**), and at 355 cm^−1^ (**d**), corresponding to the characterization peaks of MoS_2_ and WS_2_, respectively. The excitation laser is 532 nm; (**e**) AFM image of the as-grown vertical WS_2_/MoS_2_ heterostructures collected from the labeled area in figure (**a**). The inset was a corresponding height profile acquired along the gray section line. Raman single spectra (**f**) and PL single spectra (**g**) collected from different points of WS_2_/MoS_2_ LH sample, the red line and green line are corresponding to the red point and green point labeled in (**a**), respectively. (**h**) The PL spectra at a longer wavelength, and the curves were both magnified by ten times.

WS_2_/MoS_2_ VH, consisting of a MoS_2_ monolayer at the bottom and a WS_2_ monolayer on the top, can be prepared on Au foil by introducing a suitable gas flow rate of hydrogen. Figure 3a shows the typical optical image of the as-transferred stacked WS_2_/MoS_2_ VH. It can be seen that the edge length of the bottom layer MoS_2_ is ~10 μm, which is in line with those samples shown in Figure 1b and Figure 2a. In addition, the optical contrast was much larger compared to the LH shown in Figure 2a. Raman intensity mapping was conducted for the region squared in Figure 3a. Notably, the outer vertical contact area in the Raman mappings exhibit a slight color difference, attributed to the large laser spot size (∼1 µm) used in our experiment. Figure 3c,d shows the Raman intensity maps corresponding to the characteristic peak of MoS_2_ at 383 cm^−1^ and that of WS_2_ at 355 cm^−1^, respectively. The WS_2_ domain in Figure 3d is fully overlapped with the MoS_2_ domain in Figure 3c, indicating that the MoS_2_ and WS_2_ monolayers are vertically stacked rather than laterally stitched [12]. This is also verified by atomic force microscopy (AFM). Figure 3e shows the corresponding height profile acquired along the green section line, which demonstrates changes of thickness caused by different layers, corresponding to 0.75 and 0.72 nm, respectively. Furthermore, Raman and PL were used to characterize the vertical bilayer heterostructure, as shown in Figure 3f,g, respectively. The red line and green line correspond to the red point monolayer region and green point bilayer region labeled in Figure 3a. The Raman spectrum collected from the monolayer area (red point) shows only $E_{2g}^{1}$ (at 382 cm^−1^) and A_1g_ (at 401.5 cm^−1^) peaks of the MoS_2_, confirming that the bottom layer is MoS_2_. The Raman spectrum acquired from the bilayer area (green point) shows two additional peaks located at 355 and 418 cm^−1^, which are related to the $E_{2g}^{1}$ mode and the A_1g_ modes of the upside WS_2_ monolayer, respectively. Moreover, the differences between the two modes are 19.5 and 63 cm^−1^, respectively, indicating that the bottom MoS_2_ and top WS_2_ are both monolayers. The PL spectra of MoS_2_ (red curve in Figure 3g) acquired from the monolayer region (red point marked in Figure 3a) show a strong peak and a weak peak at a wavelength of 678 and 620 nm, corresponding to the A excitons and B excitons of the MoS_2_ monolayer. However, at the bilayer region (green point labeled in Figure 3a), two prominent peaks were observed at wavelengths of 638 and 678 nm, attributed to the top WS_2_ monolayer and bottom MoS_2_ monolayer [13], respectively. However, the PL intensity from the heterostructure area is much weaker than that of the MoS_2_ monolayer. Such PL quenching is attributed to the excitation-induced interlayer charge transfer across the type II heterojunction between MoS_2_ and WS_2_ [23]. As shown in Figure 3h, another PL peak with a lower intensity at 875 nm is observed in the VHs (green point marked in Figure 3a), which could originate from the interlayer excitonic transition between the minimum conduction band of MoS_2_ and the maximum valence band of WS_2_. This demonstrates the strong interlayer interactions of the WS_2_/MoS_2_ VHs.

Our results indicate that introducing H_2_ or not exhibiting such a positive effect of selectively growing WS_2_/MoS_2_ VHs or LHs on Au foil during the transition stage Ⅱ and the growth stage Ⅲ. The reason could be explained as follows: on the one hand, H_2_ can etch away excess nucleation points on the bottom layer, and the W source can reach the surface of the first as-grown MoS_2_ monolayer during the growth stage Ⅲ. Thus, uncontrolled homogeneous nucleation and muti-nucleation can be prevented effectively. On the other hand, proper H_2_ gas flow could saturate the dangling bonds on the edges of the as-grown MoS_2_ monolayer and hinder the laterally epitaxial growth [18]. The chemical potential of the edges is considerably higher compared to that of the basal planes [22,24]. Consequently, the top WS_2_ monolayer prefers to deposit on the as-grown monolayer surface rather than laterally grow in the H_2_ atmosphere. Figure S4 shows the low-magnification optical microscopic image of the as-grown WS_2_/MoS_2_ LH and VH on Au foils.

**Figure 4:**
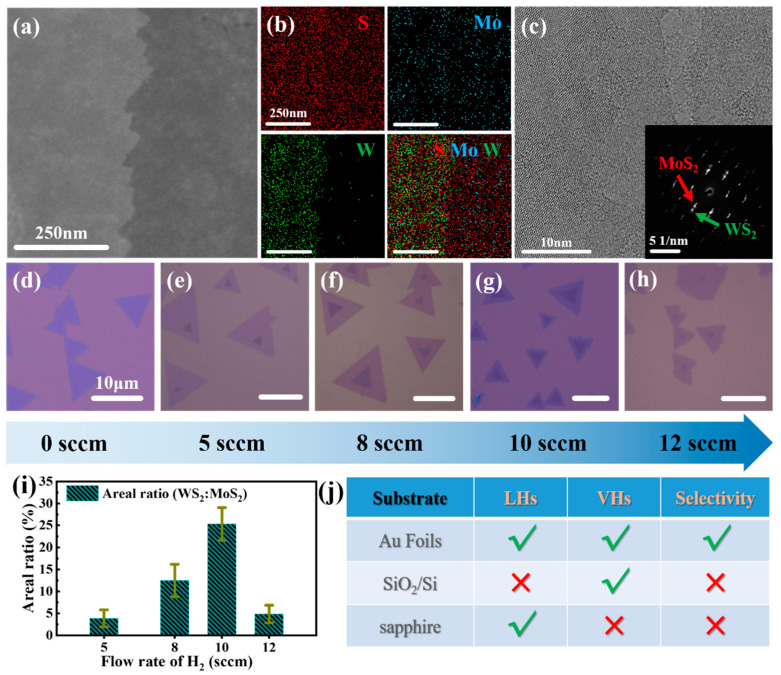
(**a**) TEM image of as-grown WS_2_/MoS_2_ VH; (**b**) the corresponding energy dispersive X-ray spectroscopy (EDS) elemental maps of the region displayed in (**a**); (**c**) HRTEM image of an as-grown WS_2_/MoS_2_ VH. The corresponding selected area electron diffraction (SAED) pattern is shown in the inset; (**d**–**h**) optical microscopic images reveal the morphology evolution of the as-transferred WS_2_/MoS_2_ heterostructures under a different gas flow rate of hydrogen; (**i**) bar chart of the WS_2_:MoS_2_ areal ratio calculated based on (**d**–**h**) under different H_2_ flow rates; (**j**) selectivity table to summarize the synthesis of LHs/VHs in various substrates.

The structure of the WS_2_/MoS_2_ VHs was examined by transmission electron microscopy (TEM). The image contrast in Figure 4a indicates the boundary between the bilayer VH (bright area) and the monolayer MoS_2_ (darker area). High-resolution TEM (HRTEM) was performed to further evaluate the qualities of as-grown WS_2_/MoS_2_ VHs, and results are presented in Figure 4c. Figure 4b is the corresponding energy dispersive X-ray spectroscopy (EDS) elemental maps of the region displayed in Figure 4a. All constituent components (Mo, W, and S) are homogenously distributed over the whole scanning range. The negligible signals of W in the MoS_2_ region excludes the cross-contamination during the CVD. The split spots in the corresponding selected area electron diffraction (SAED) pattern demonstrate the crystal structures of WS_2_ and MoS_2_, respectively, which are associated with the hexagonal symmetries of MoS_2_ and WS_2_ lattices.

Next, we investigate the effects of the H_2_ flow rates on the morphology of the WS_2_/MoS_2_ heterostructures. Figure 4d–h shows the structural evolution with an increase in the H_2_ flow rate. When no H_2_ is introduced (gas flow rate = 0 sccm), only LHs are formed on the Au substrate, and no VHs are observed (Figure 4d). When the H_2_ flow rate is increased to 5 sccm, small WS_2_ monolayer domains (edge length ~1–2 µm) are grown on top of the monolayer MoS_2_ (Figure 4e), suggesting that WS_2_/MoS_2_ VHs start to form under this condition. When the H_2_ flow rate is increased to 8 sccm, the sizes of the top WS_2_ monolayer domains are increased to ~2–5 µm, with little change in the sizes of the bottom MoS_2_ monolayer domains (~10–15 µm), and the edges of both MoS_2_ and WS_2_ domains are flat (Figure 4f). When the H_2_ flow rate is increased to ~10 sccm, as shown in Figure 4g, the sizes of top WS_2_ monolayer domains are still ~2–5 µm; however, the edges of the bottom MoS_2_ monolayer domains start to display jagged features (see in Figure S5a,b), and the domain sizes are decreased to ~5–10 µm. The formation of jagged edges indicates a change in the growth kinetics [25], which may stem from the etching effect of H_2_ [26]. With the further increase in H_2_ gas flow rate to ~12 sccm, as shown in Figure 4h, the sizes of the top layer WS_2_ domains are much smaller, and the bottom MoS_2_ layers show irregular shapes, indicating that the H_2_ induced etching dominated the growth of top and bottom layers.

Furthermore, we calculated the areal ratios of WS_2_:MoS_2_ to evaluate the morphology evolution as the H_2_ flow rate increases. The statistical results are displayed in Figure 4i. For comparison, the results of the WS_2_/MoS_2_ heterostructures grown on different substrates depending on whether or not hydrogen is supplied are presented in Figure S6. Stacked VHs are predominantly formed on SiO_2_/Si substrate when there is a supply or absence of H_2_ (see in Figure S6a,b), while the growth of WS_2_/MoS_2_ LHs is favorable on the c-sapphire substrate whether H_2_ is introduced or not (Figure S6c,d). Notably, it seems passing H_2_ will change the morphology of heterostructure domains into irregular rather than triangle-shapes. However, we do not find LHs on SiO_2_/Si or VHs on sapphire substrates by introducing H_2_, which is quite different from the results collected from Au foils. The following AFM images (Figure S6e–h) and corresponding profiles (Figure S6i–l) further support the conclusions mentioned above. The different results on metal substrate (Au) and insulating substrate (sapphire and SiO_2_/Si) can be ascribed to the TMD-substrate interactions [17]. Hence, we find it viable to dictate the growth direction (lateral or vertical) by tailoring the relationship between the adlayer material and the substrate. Finally, these substrates are classified in the selectivity table (Figure 4j) according to the synthesis results in our experimental system and the summing-up is further proof that the Au foils have selective growth advantages in 2D materials.

## 4. Conclusions

In summary, we have demonstrated one-pot CVD synthesis of 2D WS_2_/MoS_2_ heterostructures on polycrystalline Au foil. In particular, the vertical and lateral growth modes can be regulated by controlling the gas flow rate of H_2_. WS_2_/MoS_2_ LHs are obtained without H_2_ due to the good epitaxies between the two TMDs and the Au substrate. In contrast, WS_2_/MoS_2_ VHs are formed with 8–10 sccm H_2_ introduced. This could result from the decreased surface absorption energy of W species on the pre-grown MoS_2_ monolayer. Further increase in the H_2_ flow rate not only leads to the formation of VHs but also causes etching of the as-grown MoS_2_ edges. In addition, cross-contamination has been reduced via opposite directional transports of MoO_3_/WO_3_ and S vapors. Furthermore, we have shown the morphology evolution of the WS_2_/MoS_2_ VHs under different H_2_ flow rates. This work provides a feasible method that could be extended towards the growth of other 2D TMDs-based heterostructures for high performance devices.

## Data Availability

Data are contained within the article and Supplementary Materials.

## Supplementary Materials

The following supporting information can be downloaded at: https://www.mdpi.com/2079-4991/12/10/1696/s1. Figure S1. Typical optical microscopic images of monolayer MoS_2_ and WS_2_ domains grown on different substrates. (a) MoS_2_ grown on SiO_2_/Si at 750 °C for 20 min. (b) WS_2_ grown on SiO_2_/Si at 850 °C for 15 min. (c) MoS_2_ grown on c-sapphire at 750 °C for 20 min. (d) WS_2_ grown on c-sapphire at 850 °C for 15 min. Figure S2. Schematic diagrams of the growth of WS_2_/MoS_2_ LHs on Au foil and wet transfer from the Au foil onto a SiO_2_/Si substrate. Figure S3. (a) Optical microscopic image of the CVD grown MoS_2_/WS_2_ LH on Au foil. (b) XPS spectrum of the heterojunction in (a). (c–f) XPS spectra of Au 4f (c), W 4f (d), Mo 3d (e) and S 2p (f) orbitals, respectively. Figure S4. Low-magnification optical microscopic images of the as-grown (a) WS_2_/MoS_2_ LHs and (b) WS_2_/MoS_2_ VHs on Au foils, respectively. Figure S5. Optical microscopic images of as-growth MoS_2_/WS_2_ VHs (a) growth on Au foils and as-transferred MoS_2_/WS_2_ VH (b) on SiO_2_/Si, respectively. Figure S6. (a–d) Optical microscopic images of the synthesis LHs and VHs on SiO_2_/Si and c-sapphire substrates depending on whether or not hydrogen is supplied. (e–h) AFM images of as-growth MoS_2_/WS_2_ LH and VH domains corresponding to (a–d). (i–l) AFM profiles along green dash line in (e–h), respectively.
