# Supplementary material for: Selective Chemical Vapor Deposition Growth of WS2/MoS2 Vertical and Lateral Heterostructures on Gold Foils
Source: Nanomaterials (Basel). 2022 May 16;12(10):1696. doi: 10.3390/nano12101696 (PMC9144509; doi:10.3390/nano12101696)
Supplement: Supplementary file 1 [file nanomaterials-12-01696-s001.zip › nanomaterials-1707616-supplementary.pdf]

Supplementary Materials

Selective Chemical Vapor Deposition Growth of WS_2/MoS_2 Vertical and Lateral Heterostructures on Gold Foils

Zixuan Wang [1,2], Wenshuo Xu [2,3,*], Benxuan Li [2,4], Qiaoyan Hao [2], Di Wu [2], Dianyu Qi [2], Haibo Gan [2], Junpeng Xie [1], Guo Hong [1,5,*], and Wenjing Zhang [2,*]

1 Institute of Applied Physics and Materials Engineering, University of Macau, Avenida da Universidade, Taipa 999078, Macau; yb87822@umac.mo (Z.W.); jepsenxie@foxmail.com (J.X.)

2 International Collaborative Laboratory of 2D Materials for Optoelectronics Science and Technology of Ministry of Education, Institute of Microscale Optoelectronics, Shenzhen University, Shenzhen 518060, China; bl398@cam.ac.uk (B.L.); hqy@szu.edu.cn (Q.H.); physicswudi@163.com (D.W.); qidianyuqi@sina.com (D.Q.); ganhaibo@szu.edu.cn (H.G.)

3 Department of Physics, National University of Singapore, 2 Science Drive 3, Singapore, 117551, Singapore

4 Electrical Engineering Division, Engineering Department, University of Cambridge, 9 JJ Thomson Avenue, Cambridge, CB3 0FA, UK

5 Department of Physics and Chemistry, Faculty of Science and Technology, University of Macau, Avenida da Universidade, Taipa 999078, Macau

* Correspondence: wensxu@nus.edu.sg (W.X.); ghong@um.edu.mo (G.H.); wjzhang@szu.edu.cn (W.Z.)

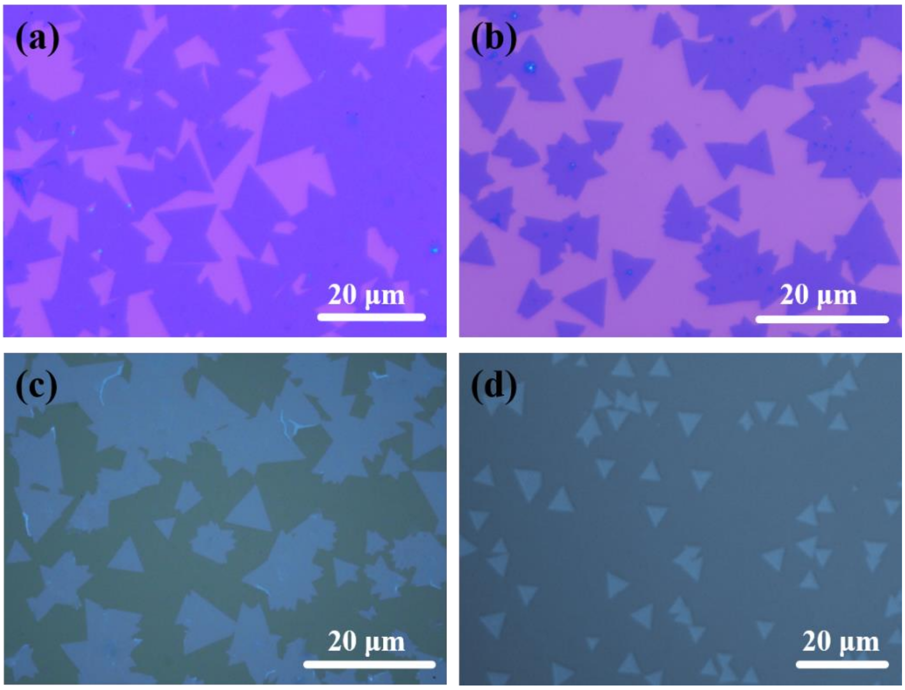

Figure S1. Typical optical microscopic images of monolayer MoS_2 and WS_2 domains grown on different substrates. (**a**) MoS_2 grown on SiO_2/Si at 750 °C for 20 min. (**b**) WS_2 grown on SiO_2/Si at 850 °C for 15 min. (**c**) MoS_2 grown on *c*-sapphire at 750 °C for 20 min. (**d**) WS_2 grown on *c*-sapphire at 850 °C for 15 min.

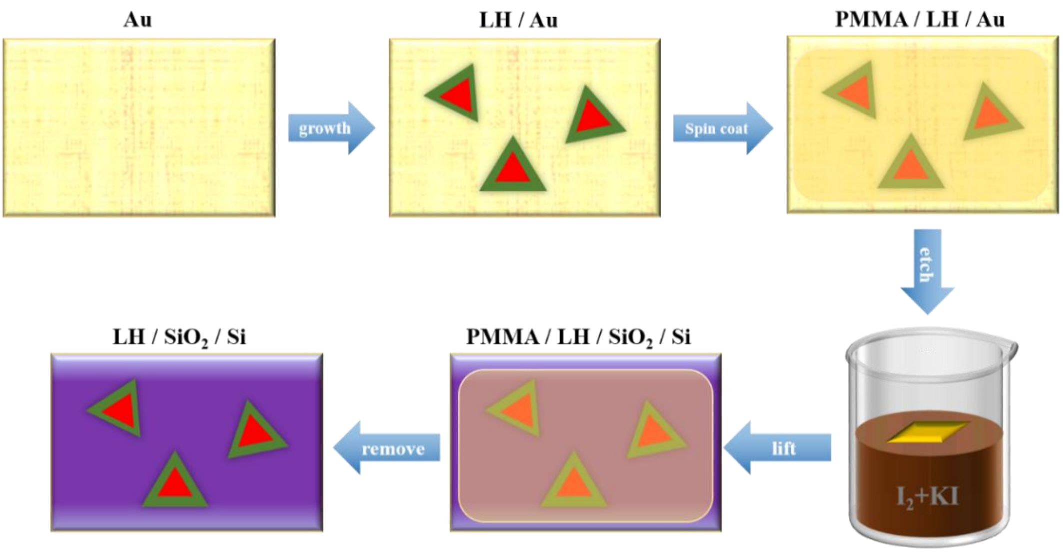

Figure S2. Schematic diagrams of the growth of WS_2/MoS_2 LHs on Au foil and wet transfer from the Au foil onto a SiO_2/Si substrate.

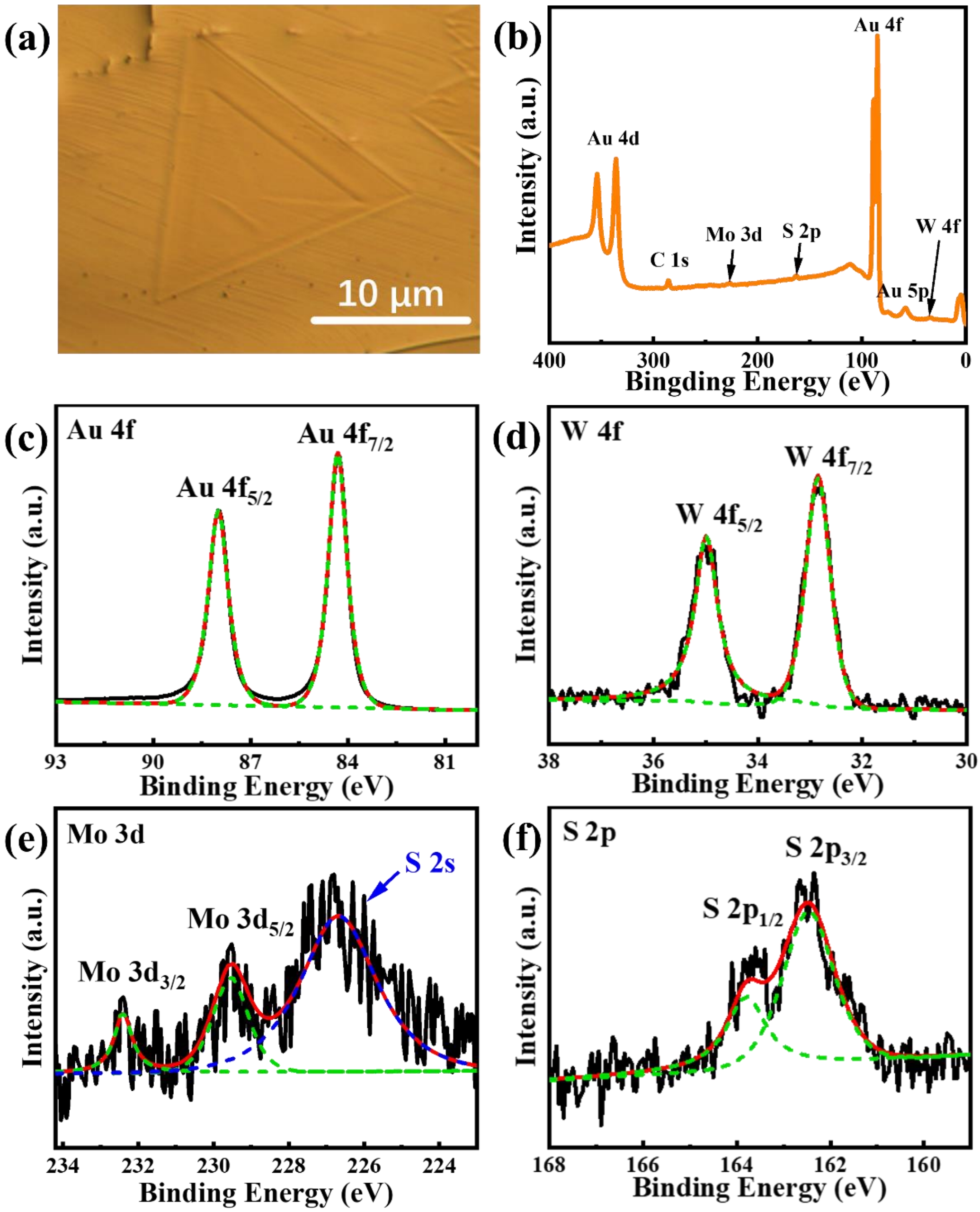

Figure S3. (**a**) Optical microscopic image of the CVD-grown MoS_2/WS_2 LH on Au foil. (**b**) XPS spectrum of the heterojunction in (**a**). (**c**–**f**) XPS spectra of Au 4f (**c**), W 4f (**d**), Mo 3d (**e**) and S 2p (**f**) orbitals, respectively.

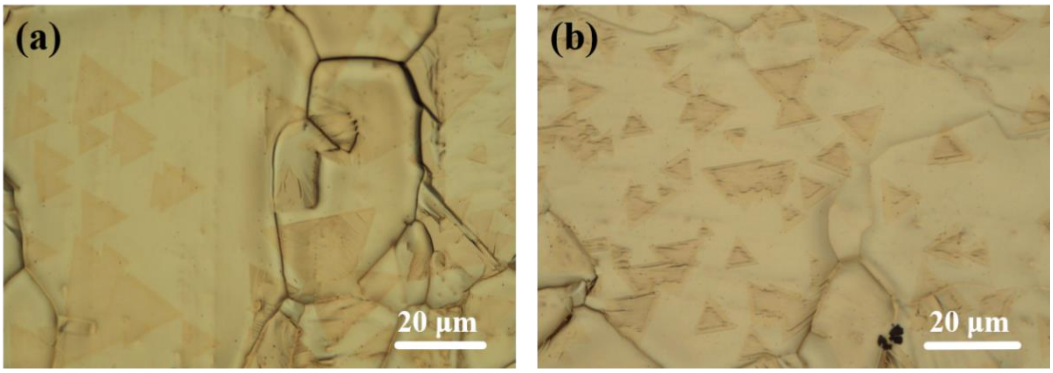

Figure S4. Low-magnification optical microscopic images of the as-grown (**a**) WS_2/MoS_2 LHs and (**b**) WS_2/MoS_2 VHs on Au foils, respectively.

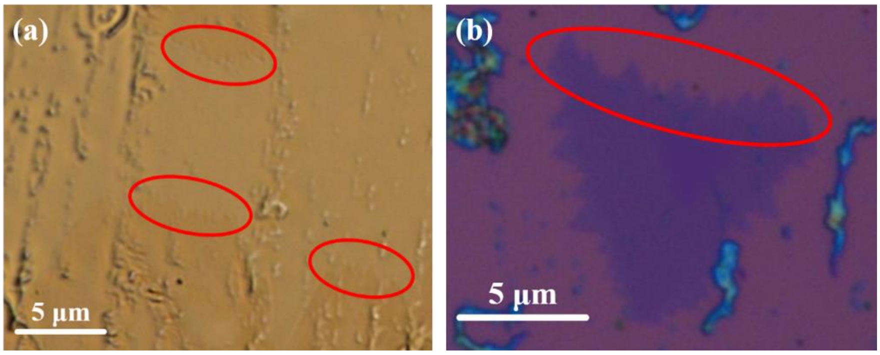

Figure S5. Optical microscopic images of as-growth MoS_2/WS_2 VHs (**a**) growth on Au foils and as-transferred MoS_2/WS_2 VH (**b**) on SiO_2/Si, respectively.

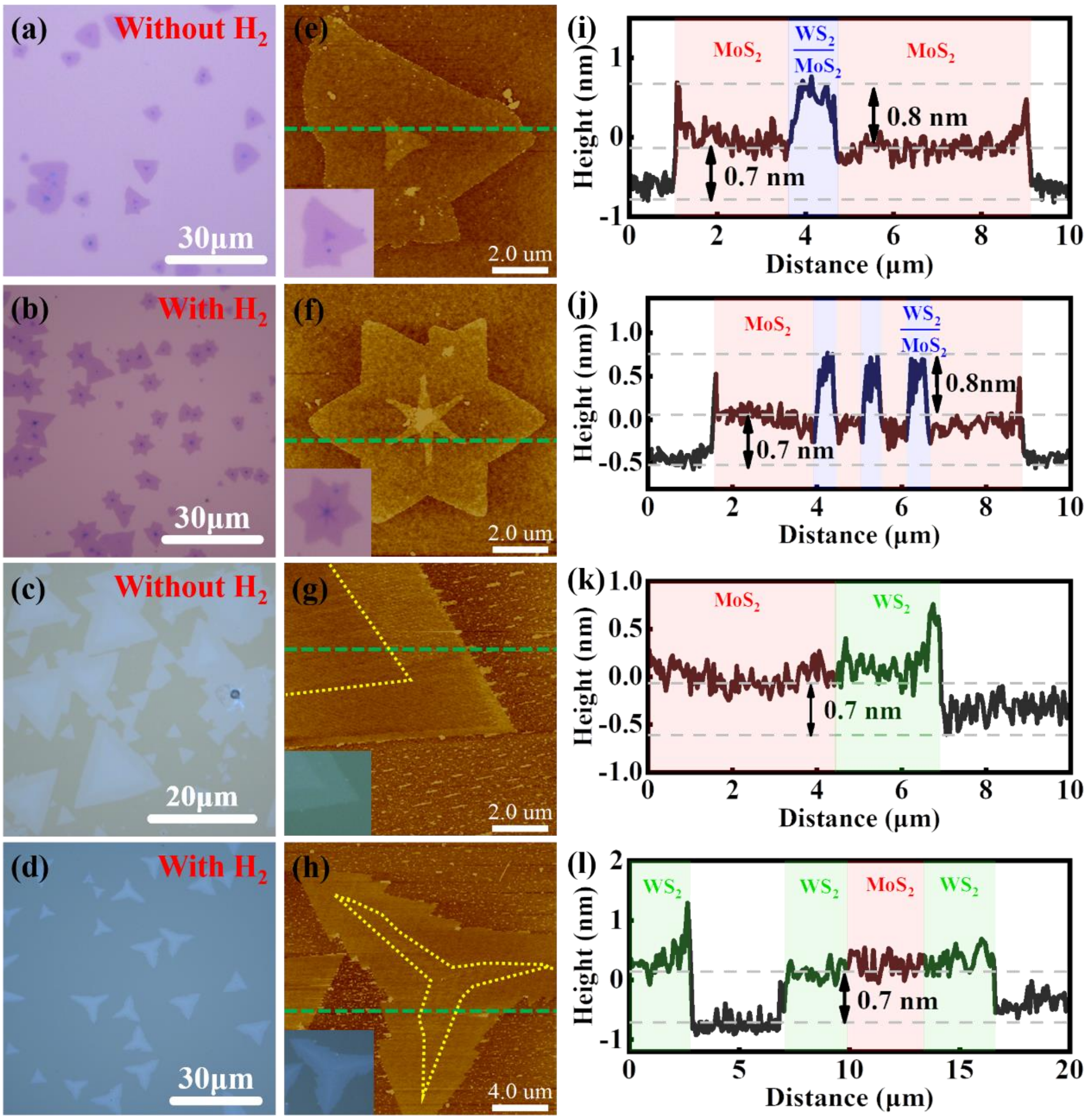

Figure S6. (**a**–**d**) Optical microscopic images of the synthesis LHs and VHs on SiO_2/Si and c-sapphire substrates depending on whether or not hydrogen is supplied. (**e**–**h**) AFM images of as-growth MoS_2/WS_2 LH and VH domains corresponding to (**a**–**d**). (**i**–**l**) AFM profiles along green dash line in (**e**–**h**), respectively.
